# The *Staphylococcus aureus* Peptidoglycan Protects Mice against the Pathogen and Eradicates Experimentally Induced Infection

**DOI:** 10.1371/journal.pone.0028377

**Published:** 2011-12-01

**Authors:** Rosanna Capparelli, Nunzia Nocerino, Chiara Medaglia, Giuseppe Blaiotta, Patrizia Bonelli, Domenico Iannelli

**Affiliations:** 1 Faculty of Biotechnology, University of Naples “Federico II”, Naples, Italy; 2 Faculty of Agriculture, University of Naples “Federico II”, Naples, Italy; 3 Istituto Nazionale per i Tumori, Fondazione G Pascale, Naples, Italy; Fundació Institut d'Investigació en Ciències de la Salut Germans Trias i Pujol. Universitat Autònoma de Barcelona. CIBERES, Spain

## Abstract

*Staphylococcus aureus*, in spite of antibiotics, is still a major human pathogen causing a wide range of infections. The present study describes the new vaccine A170PG, a peptidoglycan-based vaccine. In a mouse model of infection, A170PG protects mice against a lethal dose of *S. aureus*. Protection lasts at least 40 weeks and correlates with increased survival and reduced colonization. Protection extends into drug-resistant (MRSA or VISA) and genetically diverse clinical strains. The vaccine is effective when administered - in a single dose and without adjuvant - by the intramuscular, intravenous or the aerosol routes and induces active as well as passive immunization. Of note, A170PG also displays therapeutic activity, eradicating staphylococci, even when infection is systemic. Sustained antibacterial activity and induction of a strong and rapid anti-inflammatory response are the mechanisms conferring therapeutic efficacy to A170PG.

## Introduction

*S aureus* has developed a successful strategy of evading the human immune system, entailing the accumulation of an impressive array of virulence factors. It can colonize several niches of the human body and cause life-threatening diseases, such as pneumonia, osteomyelitis, septicaemia and endocarditis. *S. aureus* infects immunocompromised patients as well as patients without apparent risk factors [1]. About 30% of the human population carries *S. aureus* [2], a circumstance testifying the ability of this pathogen to modulate its virulence and to colonize the human host.

Strain differences in the resistance to *S. aureus* infection have been demonstrated in mice [3]. There is suggestive evidence that individual differences in the defence against *S. aureus* might also exist in humans. A polymorphism in the TIR domain of the human TLR2 gene has been reported to be associated with *S. aureus* susceptibility [4], although this association was not confirmed in a later study [5]. In addition to the capacity to cause diverse and serious diseases, *S. aureus* also displays an extraordinary potential to develop antimicrobial resistance [6]. The last decade has witnessed the emergence and rapid spread of community-associated and antibiotic-resistant strains [1]. In this context, the development of an effective vaccine appears particularly urgent.

The present article exploits the use of *S aureus* peptidoglycan (PG) as a potential vaccine. PG, a linear polymer of repeating -1-4-linked N-acetylglucosamine and N-acetylmuramic acid, accounts for approximately 50% in weight of the cell wall of Gram-positive bacteria, enabling them to resist osmotic pressure [7]. The *S. aureus* PG is recognized by the host nucleotide-binding oligomerization domain (Nod) 1 and Nod 2 [7] intracellular receptors and is involved in the activation of the complement [8], cell-mediated immunity [9] and opsonization [8]. PG is present on all the *S. aureus* bacterial strains, is exposed on the cell wall and can thus sense the external environment [8]. These features make it attractive as a vaccine.

This paper presents evidence that the PG-based vaccine A170PG, administered by the intramuscular, intravenous or aerosol routes in a single dose and without adjuvant, protects mice against an otherwise lethal dose of *S. aureus*. Protection lasts at least 40 weeks and extends to *S. epidermidis* and *L. monocytogenes*. The serum from vaccinated mice protects naïve mice against a lethal dose of *S. aureus*. Remarkably, the vaccine, administered to already infected mice, eradicates infection in one month.

## Results

### Peptidoglycan protects mice against *S. aureus*

The cell wall (CW) from the *S. aureus* strain A170 was extracted with trichloroacetic acid [10] and preliminarily purified with concanavalin A (ConA) -Agarose. Two groups of mice were then immunized with the ConA negative (ConA^-^CW) or the ConA positive (ConA^+^CW) fraction of the CW, respectively. Two weeks later the mice were challenged with an otherwise lethal dose of *S*. *aureus* A170. The mice immunized with the ConA^+^CW fraction survived all (10/10), while those immunized with the ConA^-^CW fraction died all (10/10) (Table 1; experiment 1; P: <0.0001). Following heat treatment (15 min at 100°C), the ConA^+^ fraction conserved its protective activity intact, a result which makes unlikely that protection was afforded by a glycoprotein with affinity for ConA and co-purified with the CW (Table 1; experiment 1; P: <0.0001).

**Table 1:** PG is the protective component of the ConA^+^ CW fraction.

			Interval Treatment-challenge: weeks (w)/ hours (h)	Challenge^a^		
Exp	Dose/mouse	Treatment		Pathogen	Survival	P-value^b^
1	15 µg	ConA^+^ CW	2 w	A170	10/10	<0.0001
	15 µg	ConA^-^ CW	2 w	A170	0/10	
	15 µg	Heat-treated ConA^+^ CW	2 w	A170	10/10	<0.0001
				A170	0/10	
2	10 µl	Non-absorbed ConA^+^ CW Antibodies	24 h	A170	10/10	<0.0001
		PG-Absorbed ConA^+^ CW Antibodies	24 h	A170	0/10	
3	15 µg	Lysozyme-Digested ConA^+^ CW	2 w	A170	0/10	1
	15 µg	Lysostaphin- Digested ConA^+^ CW	2 w	A170	0/10	1
4	15 µg	sPG	2 w	A170	10/10	<0.0001
				A170	0/10	

a 10^8^ CFU/mouse.

b Kaplan-Meier test.

**Figure 1:**
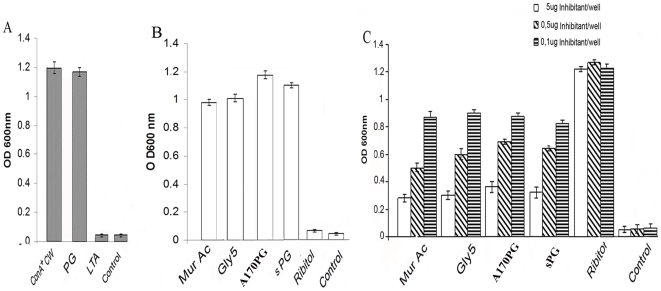
The component of the A170PG vaccine affording protection is peptidoglycan. A. The serum from mice immunized with A170PG reacts with the PG, but not with LTA, excluding that A170PG contains LTA. B. The serum from mice immunized with A170PG binds the homologous antigen (A170PG); the sPG (deprived of any CW component); Gly_5_ and muramic acid (MA), both unique component of PG. The same serum does not bind ribitol, specific to the teichoic acid. This result excludes that A170PG contains teichoic acid. C. Binding of the serum from mice immunized with A170PG to the homologous antigen (A170PG) immobilized on the plate is quantitatively inhibited by muramic acid (MA), Gly_5_, sPG, A170PG, but not ribitol.

Mindful of drawbacks often associated with *S. aureus* CW purification by chemical methods [11], [12], the identification of the ConA^+^CW component protecting mice against *S. aureus* was pursued by serological and enzymatic methods. Using as antigens the ConA^+^CW fraction, peptidoglycan (PG) and lipoteichoic acid (LTA) from *S. aureus*, the serum from mice immunized with the ConA^+^CW preparation was shown to react with the homologous antigen ConA^+^CW (as expected), with PG, but not with LTA (Figure 1, panel A). The test provided preliminary evidence that PG, but not LTA, is present in the ConA^+^CW preparation. The absence of LTA is not surprising since presumably removed by the trichloroacetic acid used to extract the CW [11].

The serum from mice immunized with the ConA^+^CW preparation protects naive mice from a lethal dose of A170. The same serum lost this property if first absorbed with PG (Table 1; experiment 2; P: <0.0001). This result is the first line of evidence that PG is the component affording protection.

The ConA^+^CW preparation was digested with lysozyme or lysostaphin. The enzyme-digested preparations were then tested to see whether they conserved the capacity to protect mice challenged with a lethal dose of the A170 strain. Digestion with lysozyme or lysostaphin destroyed the ability of ConA^+^CW to immunize mice (Table 1; experiment 3; P: 1). Given that these enzymes both hydrolyze specifically the PG [11], [13], the result provides further evidence that PG is indeed the ConA^+^CW component conferring protection against *S. aureus*.

The *S. aureus* strain A170 was grown in the presence of penicillin G (5 µg/ml). When the culture reached the stationary phase, the soluble PG (sPG) was isolated from the medium by ConA-Sepharose affinity chromatography, tested for reactivity with ConA^+^CW antibodies (Figure 1, panel B) and used to immunize mice. Challenged two weeks later with a lethal dose of A170, the immunized mice survived all (10/10) (Table 1; experiment 4; P: <0.0001). Since sPG is deprived of any CW component [11], the experiment provides compelling evidence that the protection observed in this experiment is afforded by PG.

Pentaglycine (Gl_5_) is an epitope unique to staphylococcal PG [14], [15] while muramic acid (MA) and ribitol (RBT) are monosaccharides unique to PG and teichoic acid (TA), respectively [11], [15]. Gly5, MA and RBT were therefore ideal reagents to confirm the presence of PG and the absence of TA in the ConA^+^CW preparation. In a direct ELISA assay, Gly_5_ and MA bound to the ConA^+^CW antibodies, while RBT did not (Figure 1, panel B); similarly, in a competition test only Gly_5_ and MA, along with PG, inhibited the binding of ConA^+^CW antibodies to the homologous antigen (Figure 1, panel C).

Taken together, the above experiments offer stringent and independent lines of evidence that the component of ConA*^+^*CW conferring protection against *S. aureus* infection corresponds to the PG; this component will be referred to as A170PG.

### A170PG prevents *S. aureus* infection

Protection experiments with A170PG were extended to a larger number of animals. To present the results more effectively, the details of the experiments described in this and the following sections are shown in the relevant tables or figure legends. Mice were immunized with A170PG and two weeks later infected with a lethal dose of *S. aureus* A170. At the time of infection, the antibody titre against A170PG was 10^−4^–10^−5^. The vaccinated mice survived (20/20), while control (non-vaccinated) mice died (20/20) within 4–5 days (Table 2; experiment 1; P: <0.0001). Four days after challenge, the kidneys of the A170PG-treated animals displayed significantly fewer CFU (log_10_ of CFU number: 4.7±3.6; P: <0.0001) compared to the kidneys of the control mice. Inspected four weeks after challenge, the kidneys of the vaccinated mice were sterile (Table 2; experiment 1–2). When the interval between vaccination and challenge was extended to 40 weeks, A170PG was still protective (Table 2; experiment 2; P: <0.0001).

**Table 2:** A170PG, administered by the intramuscular route at 15 µg/mouse, protects mice against *S. aureus* infection.

Exp	Vaccination		Interval vaccination/challenge (weeks)	Challenge dose (CFU/mouse)	Pathogen	Survival	P- value a	CFU/g kidneys (mean±SD; log_10_)
	Vaccine Dose (µg/mouse)							
1	A170PG	15	2	10^8^	A170	20/20	<0.0001	0^b^
				10^8^	A170	0/20		7.4±7
2	A170PG	15	40	10^8^	A170	20/20	<0.0001	0^b^
				10^8^	A170	0/20		7.3±6.7

a Kaplan-Meier test.

b CFU counted at 4 weeks from challenge.

Next experiments were aimed at optimizing the dose of the vaccine. The smallest dose of A170PG tested – 3 µg/mouse – was fully protective and safe, even when administered intravenously (Table 3; experiments 1 and 2; P: <0.0001; Figure S2). The experiments to be described were all carried out using the dose of 3 µg/mouse.

**Table 3:** A170PG, administered by the intramuscular or the intravenous routs at 3 µg/mouse, protects mice against *S. aureus*.

Exp	Vaccination		Route	Challenge dose (CFU/mouse)	Pathogen	Survival	P- value a	CFU/g kidneys (mean±SD; log_10_)
	Vaccine Dose (µg/mouse)							
1	A170PG	3	Im^b^	10^8^	A170	10/10	<0.0001	0^c^
				10^8^	A170	0/10		7.2±6.1
2	A170PG	3	Iv^d^	10^8^	A170	10/10	<0.0001	0^c^
				10^8^	A170	0/10		7.4±6.3

a Kaplan-Meier test.

b Intramuscular.

c CFU counted at 4 weeks from challenge.

d Intravenous.

### A170PG is broadly protective

The efficacy of the vaccine was also tested against 28 heterologous strains of *S. aureus*, 26 of which derived from patients with staphylococcal infections. Strains represent different genotypes, as assessed by several approaches (Table S1). Mice were vaccinated intramuscularly with 3 µg of A170PG and two weeks later were infected by the same route with a lethal dose of one of the bacterial strains. The difference in survival between vaccinated and control mice at four weeks from challenge was statistically significant in each case (Table S2). At time of death (4–5 days from infection) the kidney colonization levels among control mice ranged from 7.1±6.3 to 7.8±6.1 (log_10_ of CFU number). At four weeks from challenge the kidneys of vaccinated mice were sterile. Remarkably, the vaccine was effective against the methicillin-resistant (MRSA) strains A174, A175, RIMD31092 and the vancomycin-intermediate (VISA) strain A176 (Table S2). A170PG also protected mice against the challenge with a lethal dose of the Gram-positive bacteria *S. epidermidis* or *L. monocytogenes* (surviving mice: 10/10) (Table S2), a result concurring with the broad cross-reaction displayed by the PG preparations from *S. aureus, S. epidermidis and L. monocytogenes* (Figure S1) and the evidence that PG is highly conserved among Gram positive bacteria [16].

### The A170PG antibodies protect against *S. aureus* infection in vivo

Two groups of mice were injected intramuscularly with protein A-purified Ig from normal mouse serum or from the serum of mice immunized with A170PG (10 µl at 50 µg/ml diluted 1∶10 with sterile saline/mouse). The next day, the mice were challenged with a lethal dose of *S. aureus* A170. Survival was monitored for seven days. In two independent experiments, the serum from mice immunized with A170PG antibodies protected mice from death (20/20) (Table 4; experiments 1–2; P: <0.0001). The same serum-given at the dose of 20 µl/mouse - killed 20 out of 20 mice (Table 4; experiments 3–4; P: 1). Absorption of A170PG antibodies (10 µl at 50 µl/ml diluted 1∶10 with saline) with rat anti-total mouse immunoglobulin (5 µl at 100 µg/ml) or with A170PG (5 µl at 100 µg/ml) abrogated the protective effect of the serum (Table 4; experiments 5–6; P: 1). The above experiments demonstrate that protection afforded by the A170PG antibodies is specific (is removed by rat anti total mouse immunoglobulin or the homologous antigen); second, A170PG antibodies can be protective or detrimental, depending upon the dose being given.

**Table 4:**
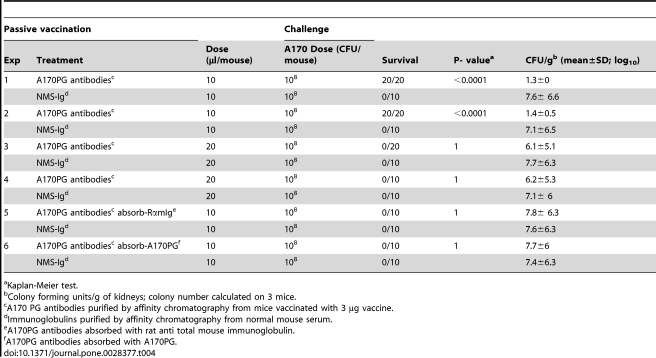
A170PG antibodies protect mice against *S. aureus* strain A170.

a Kaplan-Meier test.

b Colony forming units/g of kidneys; colony number calculated on 3 mice.

c A170 PG antibodies purified by affinity chromatography from mice vaccinated with 3 µg vaccine.

d Immunoglobulins purified by affinity chromatography from normal mouse serum.

e A170PG antibodies absorbed with rat anti total mouse immunoglobulin.

f A170PG antibodies absorbed with A170PG.

### The A170PG vaccine eradicates lung infections

*S. aureus* is one of the most common causes of pneumonia [17] and one of the bacterial pathogens most frequently detected in the respiratory secretions of patients with cystic fibrosis [18]. To explore the therapeutic potential of A170PG against lung infection, groups of mice were infected via aerosol with the A170 bacteria. Further, to ascertain whether the bacterial load influenced the result, three different infection regimens were used (10^6^, 10^7^ or 10^8^ CFU/mouse). When visibly sick (3–14 days from infection, depending upon the infection regimen used), the mice were treated with A170PG via aerosol (3 µg in 50 µl/mouse). The difference in survival between treated and untreated groups was statistically significant in each case (Table 5; P: 0.0001 or <0.0001). Remarkably, the lung colonization level was greatly reduced at one week after treatment and infection eradicated in 30 days (Table 5); by this time, in addition to the lungs, also the liver, kidneys and spleen of the treated mice were free from *S. aureus* (Table S3).

**Table 5:** Aerosol administration of A170PG vaccine (3 µg/mouse) eradicates the *S. aureus* lung infection.

	Infection regimen	Interval infection treatment (days)	Colonization level (CFU±SD; log_10_) at time of:			Colonization level (CFU±SD; log_10_) at indicated days from treatment^b^			Survival	P-value ^a^
Mice			Infection ^b^	Treatment ^b^	Death^b^					
						7	14	30		
Treated	10^8^	3	5.3±5.0	6.8±6.4		4.7±3.3	2.6±1.6	0	9/10	0.0001
Untreated	10^8^		5.4±5.1		7.2±5.8				0/10	
Treated	10^7^	9	4.5±4.3	6.7±6.3		4.7±3.6	2.3±1.5	0	10/10	<0.0001
Untreated	10^7^		4.4±4.2		7.3±6.1				0/10	
Treated	10^6^	14	3.7±3.3	6.8±5.6		4.6±3.5	2.3±1.7	0	10/10	<0.0001
Untreated	10^6^		3.8±3.2		7.2±5.3				0/10	

a Kaplan-Meier test.

b Values calculated on 3 mice.

### Protective mechanisms elicited by the A170PG vaccine

**Figure 2:**
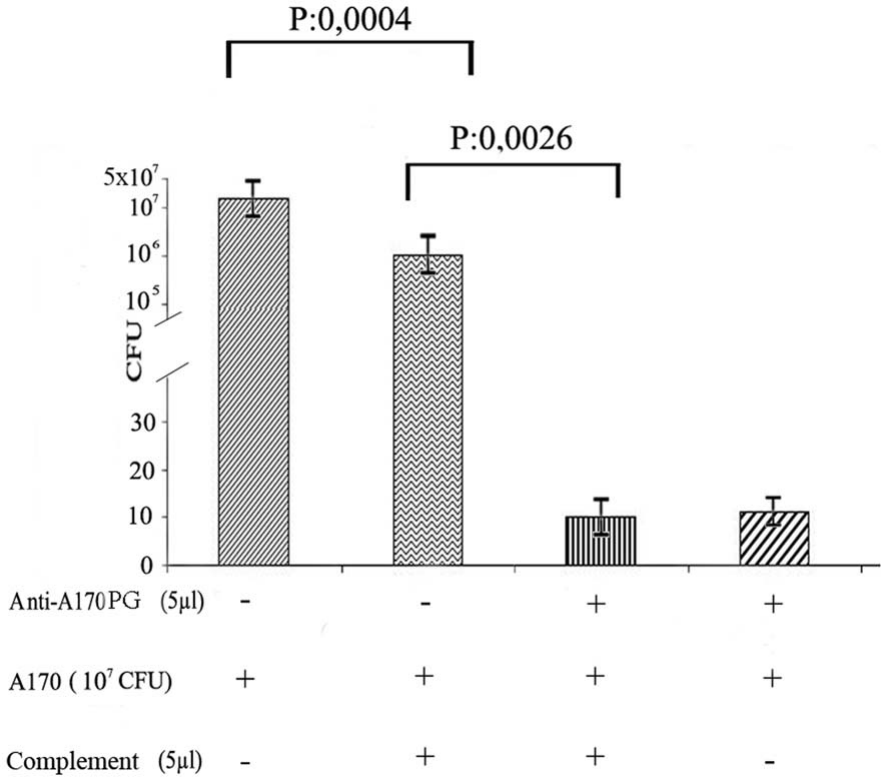
A170PG antibodies display strong bactericidal activity also in the absence of complement. A170 bacteria (10^7^ CFU/100 µl TSB medium) were incubated (1 h, 37°C) alone, with complement (5 µl), with purified A170PG antibodies (5 µl), or with complement (5 µl) and antibodies (5 µl). Bacteria were cultured on agar plates and CFU counted. Each histogram represents the results (mean±SD) of 3 independent experiments.

**Figure 3:**
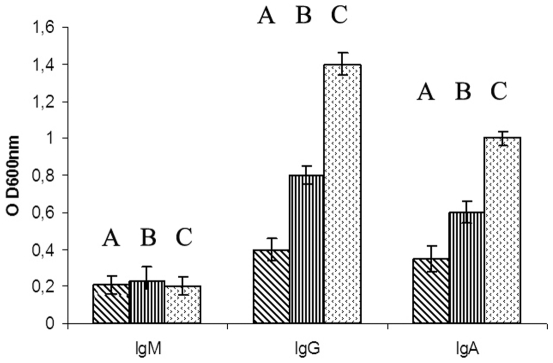
Upon receiving a second dose of the A170PG vaccine, mice undergo antibody isotype switch (from IgM to IgG or IgA) and enhanced antibody production. Mice before receiving the boost, 1 week and 2 weeks after (A–C). The time interval between the first and the second dose of vaccine was 40 weeks. Each histogram represents the results (mean±SD) from 3 mice. Conditions of the assay: A170PG (1.5 µg in 50 µl/well); pooled serum from A170PG vaccinated mice (diluted 10^−3^: 50 µl/well); rat anti mouse-IgG, -IgA or –IgM (diluted 10^−3^: 50 µl/well); peroxidase substrate (100 µl/well).

**Figure 4:**
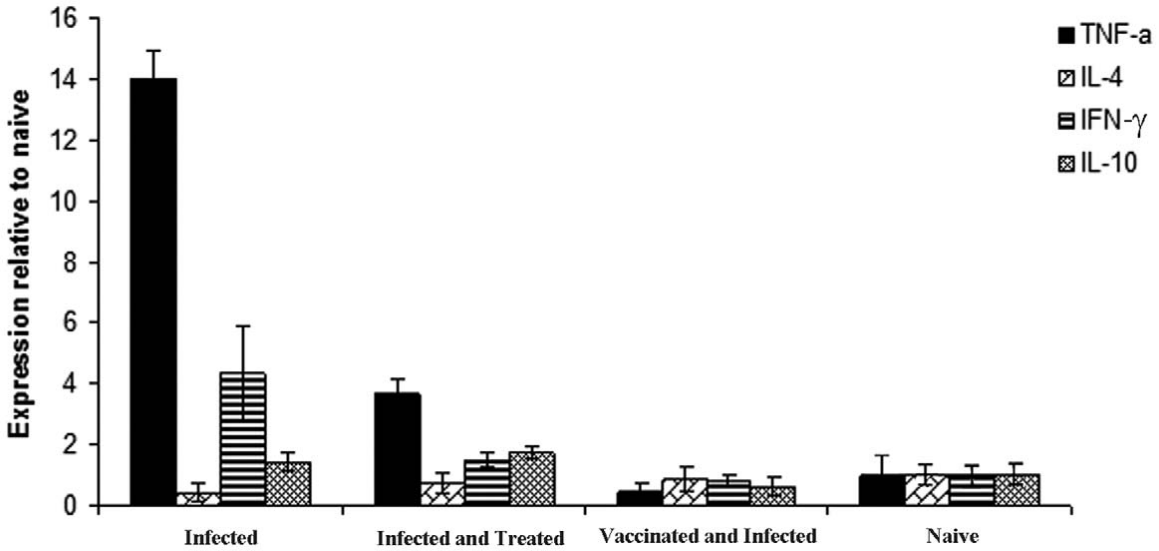
A170PG dampens the inflammatory response when used to cure or prevent *S. aureus* infection in mice. Comparison of the pro-inflammatory (TNF-α and INF-γ) and anti-inflammatory (IL-4 and IL-10) cytokines between groups of mice. Infected vs Vaccinated mice: INF-γ, P: 0.01; TNF-α, P: <0.0001; IL-4, P: 0.19; IL-10, P: 0.03. Infected vs Infected and then Treated mice: INF-γ, P: 0.03; TNF-α, P: <0.0001; IL-4, P: 0.6; IL-10, P:0.03.

**Figure 5:**
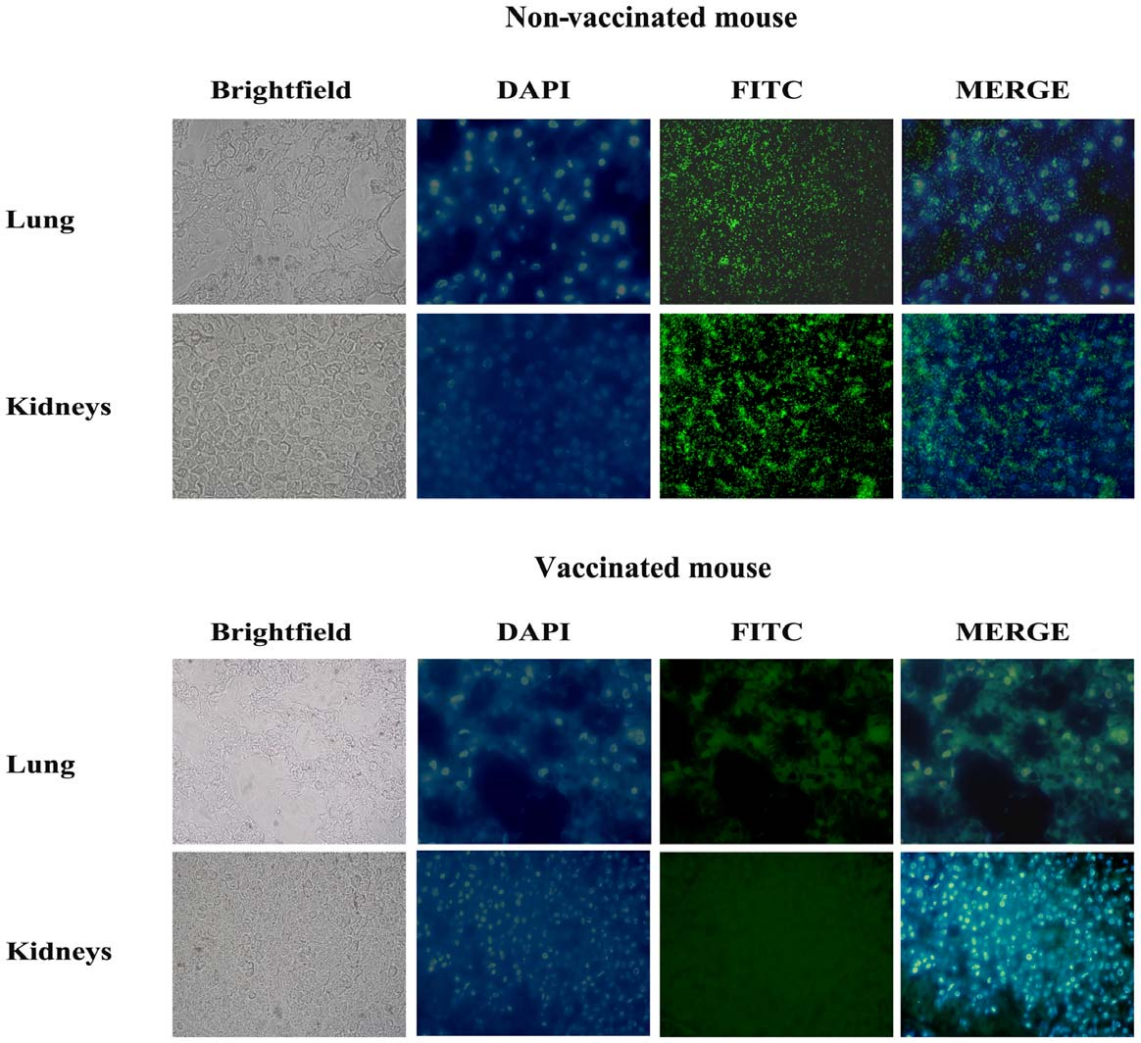
A170PG vaccination inhibits mouse organ colonization by *S. aureus*. Bacteria colonized lungs and kidneys from non-vaccinated mice, but not the same organs from vaccinated mice. Sections from vaccinated and non-vaccinated mice were incubated (2 h) with Dapi (10 µl at 0.5 µg/ml), fluorescein isothiocyanate (10 µl at 0.5 µg/ml) or neither.

Vaccines generally work by inducing antibodies with bactericidal activity, triggering a secondary response and preventing the inflammation that follows infection. The A170 bacteria were incubated (2 h at 37°) with (a) anti-A170WTA purified antibodies alone, (b) complement alone or (c) with both antibodies and complement. Bacteria were then plated and the CFU counted. Antibodies alone exerted a bactericidal activity significantly greater than that of the complement; when antibodies and complement were used in combination, complement did not enhance the bactericidal activity of the antibodies (Figure 2). Also, mice which received a boost dose of vaccine at 40-week distance from the first, displayed the main features of the vaccine-induced B cell memory: switch from the IgM to the IgG or IgA isotypes and rapid antibody production (Figure 3). Mice, vaccinated and 2 weeks later infected, displayed a cytokine profile very similar to that of naive mice; instead, the mice infected without being first vaccinated displayed significantly higher levels of the pro-inflammatory cytokines TNA-α (P: <0.0001) and IFN-γ (P: 0.01) compared to the levels of the vaccinated mice(Figure 4). Finally, *S. aureus* colonized lungs or kidneys from non-vaccinated mice, while was unable to colonize the same organs from vaccinated mice (Figure 5).

The therapeutic activity of A170PG was also characterized by a rapid reduction of the bacterial load and inflammation level of treated mice. Mice, infected and 72 h later treated with A170PG, returned free of *S. aureus* within 30 days from treatment (Table 5; Table S3) and displayed significantly lower transcription levels of the cytokines TNF-α (P: <0.0001) and IFN-γ (P: 0.03) compared to the mice which were infected, but not treated. (Figure 4).

## Discussion

In a mouse model of infection, the A170PG vaccine protects mice against a lethal dose of *S. aureus*. Protection correlates with increased survival and reduced colonization. It lasts at least 40 weeks (Table 2) and extends into genetically diverse (Tables S1) and drug-resistant (MRSA or VISA) (Table S2) clinical strains. The vaccine is effective when administered - in a single dose and without adjuvant - by the intramuscular, intravenous (Table 3) or aerosol (Table 5) routes. Significantly, A170PG eradicates the pathogen by the time infection has become systemic (Table S3). The vaccine induces active as well as passive immunization: adoptively transferred A170PG antibodies protect mice against a lethal *S. aureus* challenge (Table 4).

Several and independent experiments critically demonstrate that the biologically active component of the vaccine is the PG. First, the serum from vaccinated mice protects naive mice from a lethal dose of A170; however, this property is lost if the serum is absorbed with PG ( Table 1, experiment 2). Second, the vaccine loses the capacity to protect mice if first digested with lysozyme or lysostaphin, enzymes which hydrolyze specifically the PG (Table 1, experiment 3). Third, grown in the presence of penicillin G (which inhibits the PG incorporation into the CW), the PG accumulates in the culture medium in a soluble form (sPG) deprived of any CW component [11]; the sPG from the A170 strain effectively protects mice against *S. aureus* (Table 1, experiment 4). Fourth, the serum from mice vaccinated with A170PG recognizes two components unique of the PG: Gl_5_ and MA (Figure 1, panel B).

The results described in this study, while promising, require a critical analysis in the light of the current literature. The mouse strain used in this study (Balb/c) is highly susceptible to *S. aureus* [3]. Still, large bacterial doses are needed for infection. The model therefore does not mimic closely human staphylococcal infections, which are generally caused by a small initial inoculum. Second, unlike humans, the mouse does not have pre-existing antibodies to *S. aureus* [19]. Therefore, one must contemplate the possibility that A170PG - which works well in mice - might not work equally well in humans since it could be neutralized by pre-existing antibodies. More important, there is evidence that staphylococcal escape strategies from immune defence mechanisms are by far more efficient in humans than in mice [20].

PG shows limited serological variability [16] (Figure S1), confirmed by the capacity of A170PG to protect against *S. aureus, S. epidermidis* and *L. monocytogenes* (Table S2). PG thus represents an attractive choice as vaccine. However, previous single component vaccines have given disappointing results in humans [19]. StaphVAX (a capsular polysaccharide vaccine) in the confirmatory phase III clinical trial offered no significant protection against *S. aureus*, compared to the control group [19]. Thus, the efficacy of A170PG as single component vaccine in humans remains to be confirmed by clinical testing.

Some categories of patients (such as premature neonates and haemodialysis patients), at high risk of staphylococcal infections, do not respond adequately to immunization. These patients benefit particularly from passive immunoprophylaxis. A170PG antibodies provide passive protection, at least in mice (Table 4). In vivo, A170PG antibodies display prozone effect (see below), a property that could not be anticipated on the basis of their functions in vitro and potentially relevant to passive immunoprophylaxis against *S. aureus*. Purified A170PG antibodies were protective only when administered within a restricted range: the dose of 10 µl/mouse rescued all mice (20/20) from a lethal dose of A170 bacteria (Table 4; experiments 1 and 2); the dose of 20 µl/mouse of the same preparation instead killed all (20/20) the mice (Table 4, experiments 3 and 4). Incidentally, the observation that antibodies are not protective if used in excess is not new and in the literature the phenomenon is described with the term of “prozone effect” [21].

In a trial with AltaStaph (a pool of sera from volunteers immunized with StaphVAX), 24% of the patients treated with AltaStaph and standard therapy died, compared to 11% of those treated with the standard therapy alone [19]. The result was interpreted as evidence of the limited use of the antibodies against capsular polysaccharides [19]. The data reported above make it plausible also that failure of AltaStaph might have been caused by the use of an excess of antibodies (and concurrent prozone effect). More in general, the prozone effect perhaps deserves careful consideration when interpreting passive protection experiments.

A170PG exerts its prophylactic activity by inducing antibodies able to kill bacteria (Figure 2), inhibit organ colonization (Figure 5), evoke B cell memory (Figure 3) and an anti-inflammatory response (Figure 4). A170PG has also therapeutic capacity. The vaccine eradicates infection (even when systemic) with surprising efficacy and rapidity (Table S3; Table 5). One single dose of vaccine administered via aerosol to mice previously infected with a lethal dose of A170 rescued all the mice, while control (infected but not treated) mice all died (Table 5). In these experiments, mice were treated with A170PG when severely sick. Still, treated mice all eradicated the staphylococci within one month from treatment (Table S3; Table 5 ). In addition, A170PG induced also a strong anti-inflammatory response (Figure 4). While these results are robust evidence of the vaccine's therapeutic potential, the mechanisms responsible for the therapeutic efficacy remain conjectural. Monocytes and macrophages are the main source of pro-inflammatory cytokines [16]. At the same time they play a major role also in the antibacterial response [22], [23]. This study shows that macrophage exposure to A170PG suppresses the production of pro-inflammatory cytokines (Figure 4), but leaves the antibacterial activity intact, which culminates in the eradication of the pathogen (Table S3; Table 5). Though seemingly contradictory, these results become consistent if one assumes that PG can induce macrophage tolerance, much as LPS does [22]. Exposure to LPS inactivates the macrophage pro-inflammatory genes, while leaving inducible the antibacterial response genes; this permits the avoidance of excessive inflammation and septic shock while fighting pathogens [22]. Here it is proposed that the same might occur in the case of macrophages exposure to PG. Phrased another way, PG would dampen inflammation in the case of Gram-positive infections, just as LPS does in the case of Gram-negative infections. This hypothesis – though to be confirmed by future studies - is attractive also because it would explain why PG - a known bacterial virulence factor [8], [9] - is not harmful when used as vaccine: an excess of PG, as in this study (3–15 µg/mouse), it would induce desensitization of the downstream inflammation signalling pathways.

In conclusion, A170PG potentially can find application in the prophylaxis as well as the therapy of staphylococcal infections, a rare property for a vaccine. At the same time, one must prudently consider that A170PG has been tested in a mouse model and the mouse is not a natural host for *S. aureus*. Whether this seemingly promising vaccine will protect humans can only emerge from clinical trial.

## Materials and Methods

### Ethics Statement

This study was carried out in strict accordance with the recommendations in the Guide for the Care and Use of Laboratory Animals of the European Community. The protocol was approved by the Committee on the Ethics of Animal Experiments of the University of Naples (Permit Number: 86/609/EEC). All surgery was performed under sodium pentobarbital anesthesia, and all efforts were made to minimize suffering.

### Bacteria

The sources of the *Staphylococcus aureus* strains used in the study are shown in Table S1. The *Staphylococcus epidermidis* strain and *Listeria monocytogenes* strain were collected from patients hospitalized at the Medical School of the University of Naples. Molecular characterization of the *S. aureus* strains included analysis of the rDNA V3 spacer region [24], capsular polysaccharide [25], enterotoxin genes [26], [27], Sma 1 restriction endonuclease analysis [28] and protein A gene (*spa*) typing [29]. Clinical samples were streaked on trypticase soy agar (TSA) (Oxoid, Milan, Italy) and single colonies expanded in trypticase soy broth (TSB) (Oxoid). For in vivo and in vitro experiments, bacteria were grown in TSB at 37° C, harvested while in the exponential growth phase (OD_600_: 1.5 to 1.8), centrifuged (8×10^3^ x *g* for 10 min) and washed with saline (0.15 M NaCl). Serial 10-fold dilutions in saline were then plated on TSA. To isolate the soluble form of the peptidoglycan (sPG), the *S. aureus* strain A170 bacteria was grown in TSB in the presence of penicillin G (Biopharma, Italy; 5 µg/ml). When the culture was in the exponential growth phase, the sPG was isolated from the medium by ConA-Agarose affinity chromatography.

### ConA-Agarose affinity chromatography

CW was extracted from *S. aureus, S. epidermidis or L. monocytogenes* with trichloroacetic acid [10] and further purified with concanavalin A conjugated to agarose (ConA-Agarose; Sigma, Milan, Italy). The lectin suspension (500 µl) was centrifuged, washed with Tris-HCl buffer and the pellet suspended in 500 µl of the same buffer. CW (2.8 mg in 5 ml Tris-HCl buffer pH 7.5) was mixed with the ConA-Agarose, incubated for 1 h, centrifuged and the supernatant (ConA^-^ fraction) recovered. The pellet was washed three times with Tris-HCl buffer and the CW eluted from the matrix with 5 ml of 0.2 M α-methyl-diglucoside (Sigma) (ConA^+^CW fraction). The ConA^-^CW and ConA^+^CW fractions were desalted (Sephadex G-25), lyophilized, weighed and tested separately for the capacity to protect mice.

### Mice

Experiments were carried out on female Balb/c mice (aged 8 to 10 weeks) at the animal facility of the University of Naples. Mice infection and vaccination were carried out via the intramuscular, intravenous or aerosol routes. For intramuscular or intravenous infection, individual mice were injected with 10^8^ colony forming units (CFU)/100 µl saline. For aerosol infection, five mice were placed in a nebulizing chamber and exposed to the aerosol bacterial suspension (10^6^ to 10^8^ CFU/100 µl saline). The ConA^+^CW fraction from the *S. aureus* strains A170 was administered intramuscularly (3–15 µg in 50 µl/mouse) or by aerosol (3–15 µg/50 µl saline/mouse). Organs were dissected and weighed. Samples were homogenized in 1 ml saline and serially diluted in the same medium. CFU were evaluated by the plate count assay and expressed as CFU/g.

### Detection of ConA^+^CW antibodies

The wells of a 96-well plate (Falcon, Milan) were coated with the antigen being tested: muramic acid (MA), pentaglycine (Gly^5^), ribitol (RBT), lipoteichoic acid (LTA) (all from Sigma); ConA^+^ or ConA^-^ fractions of the CW from *S. aureus, S. epidermidis* or *L. monocytogenes*; soluble peptidoglycan (sPG). Antigens were used at 0.1–15 µg in 50 µl/well. The plate was quenched with 3% bovine serum albumin (50 µl/well; 2 h), washed with PBS, incubated overnight with purified antibodies from mice immunized with ConA^+^CW (diluted 10^−2^–10^−4^; 50 µl/well). Antibodies were purified by protein A affinity chromatography [30] and brought to 50 µg/ml. Plates were washed with PBS and incubated, in succession, with peroxidase-labelled secondary antibodies (rat anti total mouse immunoglobulin, rat anti mouse-IgG, -IgM or -IgA diluted 10^−3^ (50 µl/well; 2 h; Sigma) and peroxidase substrate (100 µl/well; 1 h; Bio-Rad, Milan). Optical density (OD) was measured at 600 nm. Samples were run in triplicate.

### Other methods

To test the biological activity of the A170PG, *S. aureus* A170 bacteria (10^7^ CFU/100 µl saline) were incubated (1 h at 37°C) with 5 µl of A170PG purified antibodies or with 5 µl of the same antibodies plus 5 µl of complement (BioMerieux, France). CFU were evaluated by the plate count assay. ConA^+^CW (0.28 mg in 500 µl Tris-HCl pH7.5) was digested with lysozyme (Sigma; 220 U/ml; 2 h) or lysostaphin (Sigma; 220 U/ml; 2 h) and then used to immunize mice (50 µl/mouse). The transcription level of mouse cytokine genes was analyzed by real-time reverse transcription PCR (RT-PCR) as described [31]. Survival rates of mice were analyzed using the Kaplan-Meier test. Bacterial counts and gene expression levels were analyzed using the paired t test (P values are two-tailed values).

## Supporting Information

Figure S1 **Cross-reaction of PG preparations from** ***L. monocytogenes*** **(A),** ***S. epidermidis*** **(B) and** ***S. aureus*** **(C).** Conditions of the ELISA assay 1.5 µg PG preparation in 50 µl/well; serum from mice immunized with A170PG diluted 10^−3^: 50 µl/well; rat anti mouse IgG diluted 10^−3^: 50 µl/well.
(TIF)

Figure S2 **Kaplan-Meier survival curves of mice vaccinated with A170PG (3 µg/mouse) by the aerosol, intramuscular or intravenous routes.** Vaccinated and control mice were challenged with 10^8^ CFU/mouse.
(TIF)

Table S1 **Molecular characterization of** ***S. aureus*** **strains.**
(DOC)

Table S2 **Broad protective activity of the A170PG vaccine.**
(DOC)

Table S3 **Aerosol administration of A170PG eradicates** ***S. aureus*** **systemic infection.**
(DOC)
