# Supplementary figures and images for: The Staphylococcus aureus Peptidoglycan Protects Mice against the Pathogen and Eradicates Experimentally Induced Infection
Source: PLoS One. 2011 Dec 1;6(12):e28377. doi: 10.1371/journal.pone.0028377 (PMC3228750; doi:10.1371/journal.pone.0028377)

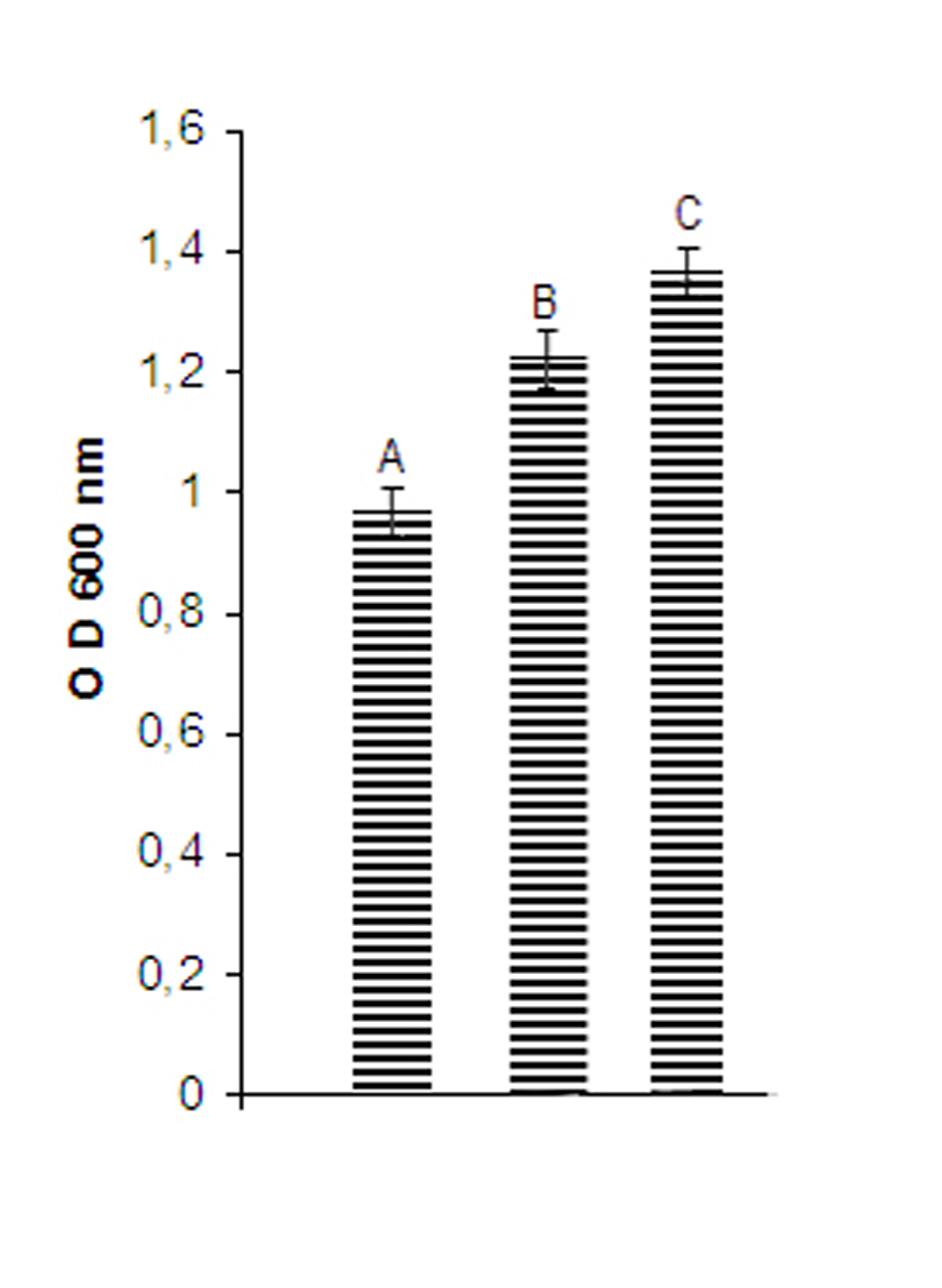

Supplement: Figure S1 — Cross-reaction of PG preparations from L. monocytogenes (A), S. epidermidis (B) and S. aureus (C). Conditions of the ELISA assay 1.5 µg PG preparation in 50 µl/well; serum from mice immunized with A170PG diluted 10−3: 50 µl/well; rat anti mouse IgG diluted 10−3: 50 µl/well. (TIF) [file pone.0028377.s001.tif]

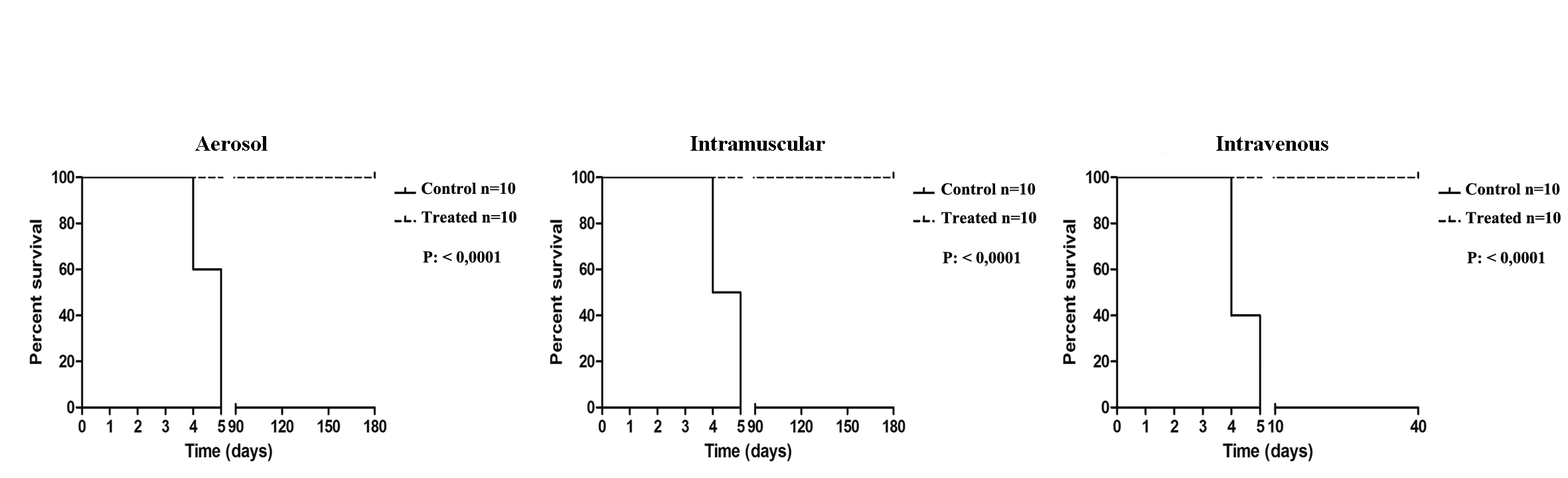

Supplement: Figure S2 — Kaplan-Meier survival curves of mice vaccinated with A170PG (3 µg/mouse) by the aerosol, intramuscular or intravenous routes. Vaccinated and control mice were challenged with 108 CFU/mouse. (TIF) [file pone.0028377.s002.tif]
