# Supplementary material for: The Staphylococcus aureus Peptidoglycan Protects Mice against the Pathogen and Eradicates Experimentally Induced Infection
Source: PLoS One. 2011 Dec 1;6(12):e28377. doi: 10.1371/journal.pone.0028377 (PMC3228750; doi:10.1371/journal.pone.0028377)
Supplement: Table S1 — Molecular characterization of S. aureus strains. (DOC) [file pone.0028377.s003.doc]

Table S1: Molecular characterization of *S. aureus* strains.

| **Strain** | **Source** | **ISR**  **Type^a^** | **Cap**  **Type^b^** | **SEs**  **Genotype^c^** | **REA-*egc***  **Type^d^** | ***spa***  **Type^e^** | ***Sma* I**  **REA-PFGE^f^** |
| --- | --- | --- | --- | --- | --- | --- | --- |
| A170^3^ | UNSM^6^ | A | 8 | egc^+^ | 4 | t6668 | A |
| A172^3^ | UNSM^6^ | A | 8 | egc^+^ | 4 | t6668 | A |
| S.a135^3^ | UNSM^6^ | A | 8 | egc^+^ | 4 | t6668 | A |
| S.a143^3^ | UNSM^6^ | A | 8 | egc^+^ | 4 | t6668 | A |
| S.a144^3^ | UNSM^6^ | A | 8 | egc^+^ | 4 | t6668 | A |
| S.a152^3^ | UNSM^6^ | A | 8 | egc^+^ | 4 | t6668 | A |
| S.a161^3^ | UNSM^6^ | A | 8 | egc^+^ | 4 | t6668 | A |
| S.a174^1,3^ | UNSM^6^ | A | 8 | egc^+^ | 4 | t6668 | A |
| S.a175^1,3^ | UNSM^6^ | A | 8 | egc^+^ | 4 | t6668 | A |
| S.a176^2,3^ | UNSM^6^ | A | 8 | egc^+^ | 4 | t6668 | A |
| S.a180^3^ | UNSM^6^ | A | 8 | egc^+^ | 4 | t6668 | A |
| S.a200^3^ | UNSM^6^ | A | 8 | egc^+^ | 4 | t6668 | A |
| S.a215^3^ | UNSM^6^ | A | 8 | egc^+^ | 4 | t6668 | A |
| S.a216^3^ | UNSM^6^ | A | 8 | egc^+^ | 4 | t6668 | A |
| S.a220^3^ | UNSM^6^ | A | 8 | egc^+^ | 4 | t6668 | A |
| S.a259^3^ | UNSM^6^ | A | 8 | egc^+^ | 4 | t6668 | A |
| S.a352^3^ | UNSM^6^ | A | 8 | egc^+^ | 4 | t6668 | A |
| S.a373^3^ | UNSM^6^ | A | 8 | egc^+^ | 4 | t6668 | A |
| S.a375^3^ | UNSM^6^ | A | 8 | egc^+^ | 4 | t6668 | A |
| S.a469^3^ | UNSM^6^ | A | 8 | egc^+^ | 4 | t6668 | A |
| S.a697^3^ | UNSM^6^ | A | 8 | egc^+^ | 4 | t6668 | A |
| DSM20231^3^ | DSMZ^7^ | B | 5 | negative | nt^11^ | t011 | nt^11^ |
| ATCC14458^3^ | ATCC^8^ | C | 5 | seb^+^ | nt^11^ | t008 | nt^11^ |
| ATCC27664^4^ | CNTS^9^ | D | 8 | negative | nt^11^ | t029 | nt^11^ |
| RIMD31092^1,3^ | CNTS^9^ | E | 5 | seb^+^, sec^+^, egc^+^ | 1 | t002 | nt^11^ |
| ATCC19095^3^ | CNTS^9^ | F | 5 | sec^+^, seh^+^, egc^+^ | 2 | t352 | nt^11^ |
| ATCC25923^3^ | ATCC^8^ | G1 | 8 | egc^+^ | 5 | t021 | nt^11^ |
| AB-8802^5^ | DSAN^10^ | G2 | 8 | egc^+^ | 6 | t021 | nt^11^ |

^a^16S-23S rDNA intergenic spacer region type

^b^Capsular polysaccharides type

^c^Staphylococcal enterotoxin genotype

^d^Restriction endonuclease analysis of the enterotoxin gene cluster (*egc*)

^e^Protein A gene typing

^f^Restriction endonuclease analysis-pulsed-field gel electrophoresis

^1^MRSA (Methicillin-resistant *S. aureus*)

^2^VISA (Vancomycin-intermediate *S. aureus*)

^3^Clinical samples

^4^Chicken tetrazzini

^5^Raw poultry meat

^6^University of Naples, School of Medicine

^7^Deutsche Sammlung fur Mikroorganismen und Zellkulturen, Braunschweig, Germany

^8^American Type Culture Collection, Rockville, Md.

^9^Centre Nationale des Toxemies a Staphylococques, Facultè de Medecine Laennee, Lyon, France (kindly provided by G. Lina)

^10^Dipartimento di Scienza degli Alimenti, Università degli Studi di Napoli Federico II

^11^Not tested
