# Supplementary material for: The Staphylococcus aureus Peptidoglycan Protects Mice against the Pathogen and Eradicates Experimentally Induced Infection
Source: PLoS One. 2011 Dec 1;6(12):e28377. doi: 10.1371/journal.pone.0028377 (PMC3228750; doi:10.1371/journal.pone.0028377)
Supplement: Table S2 — Broad protective activity of the A170PG vaccine. (DOC) [file pone.0028377.s004.doc]

Table S2. Broad protective activity of the A170PG vaccine.

| Challenging pathogen | Survival^a^ | P-value^b^ |  |
| --- | --- | --- | --- |
| S.a^c^ 135 | 10/10 | < 0.0001 |  |
| S.a 143 | 10/10 | < 0.0001 |  |
| S.a 144 | 9/10 | 0.0001 |  |
| S.a 152 | 10/10 | < 0.0001 |  |
| S.a 161 | 8/10 | < 0.0003 |  |
| S.a 169 | 10/10 | < 0.0001 |  |
| S.a A174 (MRSA)^d^ | 10/10 | < 0.0001 |  |
| S.a A175 (MRSA) | 10/10 | < 0.0001 |  |
| S.a A176 (VISA)^e^ | 8/10 | < 0.0003 |  |
| S.a 180 | 10/10 | < 0.0001 |  |
| S.a 200 | 9/10 | 0.0001 |  |
| S.a 215 | 9/10 | 0.0001 |  |
| S.a 216 | 10/10 | < 0.0001 |  |
| S.a 220 | 10/10 | < 0.0001 |  |
| S.a 259 | 10/10 | < 0.0001 |  |
| S.a 352 | 8/10 | < 0.0003 |  |
| S.a 373 | 10/10 | < 0.0001 |  |
| S.a 375 | 10/10 | < 0.0001 |  |
| S.a 469 | 10/10 | < 0.0001 |  |
| S.a 697 | 8/10 | < 0.0003 |  |
| S.e^f^ 001 | 10/10 | < 0.0001 |  |
| L.m^g^ 001 | 10/10 | < 0.0001 |  |
| DSM20231 | 10/10 | < 0.0001 |  |
| ATCC14458 | 9/10 | 0.0001 |  |
| ATCC27664 | 10/10 | < 0.0001 |  |
| RIMD31092(MRSA)^d^ | 10/10 | < 0.0001 |  |
| ATCC19095 | 10/10 | < 0.0001 |  |
| ATCC25923 | 9/10 | 0.0001 |  |
| AB-8802 | 10/10 | < 0.0001 |  |

^a^ Mice were vaccinated intramuscularly with A170PG (3µg/mouse) and two weeks later infected by the same route with a lethal dose (10^8^ CFU/mouse) of the listed pathogen. Survival rate of controls was 0/10 in each case.

^b^ Kaplan-Meier test. CFU/g among controls ranged from 1.2 x 10^7^ ± 2 x 10^6^ to 7 x 10^7^ ± 1.4 x 10^6^.

^c^ SA = *S. aureus*.

^d^ MRSA = Methicilin-resistant *S. aureus.*

^e^ VISA = Vancomycin-intermediate *S. aureus.*

^f^ S.e = *Staphylococcus epidermidis*.

^g^ L. m = *Listeria monocytogenes*.
