# Supplementary material for: The Staphylococcus aureus Peptidoglycan Protects Mice against the Pathogen and Eradicates Experimentally Induced Infection
Source: PLoS One. 2011 Dec 1;6(12):e28377. doi: 10.1371/journal.pone.0028377 (PMC3228750; doi:10.1371/journal.pone.0028377)
Supplement: Table S3 — Aerosol administration of A170PG eradicates S. aureus systemic infection. (DOC) [file pone.0028377.s005.doc]

| Organ | Mice | Infection regimen | Colonization level (CFU ± SD; log_10_) at death^a^ | Colonization level (CFU ± SD) at indicated days from treatment | | | Survival | P-value^b^ |
| --- | --- | --- | --- | --- | --- | --- | --- | --- |
|  |  |  |  | 7 | 14 | 30 |  |  |
| Kidneys | Treated | 10^8^ |  | 4.3 ± 3.3 | 2.3 ± 1.6 | 0 | 8/10 | 0.0003 |
|  | Untreated | 10^8^ | 7.4 ± 6.5 |  |  |  |  |  |
|  |  |  |  |  |  |  |  |  |
|  | Treated | 10^7^ |  | 4.4 ± 3.3 | 2.2 ± 1.3 | 0 | 10/10 | < 0.0001 |
|  | Untreated | 10^7^ | 7.5 ± 6.1 |  |  |  |  |  |
|  |  |  |  |  |  |  |  |  |
|  | Treated | 10^6^ |  | 4.3 ± 3.5 | 2.1 ± 1.5 | 0 | 10/10 | < 0.0001 |
|  | Untreated | 10^6^ | 7.2 ± 6.6 |  |  |  |  |  |
|  |  |  |  |  |  |  |  |  |
| Spleen | Treated | 10^8^ |  | 3.7 ± 2.3 | 2.3 ± 1.5 | 0 | 8/10 | 0.0003 |
|  | Untreated | 10^8^ | 4.6 ± 3.5 |  |  |  |  |  |
|  |  |  |  |  |  |  |  |  |
|  | Treated | 10^7^ |  | 3.7 ± 2.6 | 2.4 ± 1.6 | 0 | 10/10 | < 0.0001 |
|  | Untreated | 10^7^ | 4.6 ± 3.4 |  |  |  |  |  |
|  |  |  |  |  |  |  |  |  |
|  | Treated | 10^6^ |  | 3.4 ± 2. | 2.2 ± 1.6 | 0 | 10/10 | < 0.0001 |
|  | Untreated | 10^6^ | 4.4 ± 3 |  |  |  |  |  |
|  |  |  |  |  |  |  |  |  |
| Liver | Treated | 10^8^ |  | 3.3 ± 2.4 | 2.3 ± 1.7 | 0 | 8/10 | 0.0003 |
|  | Untreated | 10^8^ | 5.1 ± 4.5 |  |  |  |  |  |
|  |  |  |  |  |  |  |  |  |
|  | Treated | 10^7^ |  | 3.5 ± 2.6 | 2.2 ± 1.6 | 0 | 10/10 | < 0.0001 |
|  | Untreated | 10^7^ | 5.1 ± 4.3 |  |  |  |  |  |
|  |  |  |  |  |  |  |  |  |
|  | Treated | 10^6^ |  | 3.4 ± 2.3 | 2.1 ± 1.3 | 0 | 10/10 | < 0.0001 |
|  | Untreated | 10^6^ | 5 ± 4.4 |  |  |  |  |  |

^a^ Values calculated on 3 mice.

^b^Kaplan-Meier test.
